# Salivary Immunoglobulin A Secretion and Polymeric Ig Receptor Expression in the Submandibular Glands Are Enhanced in Heat-Acclimated Rats

**DOI:** 10.3390/ijms21030815

**Published:** 2020-01-27

**Authors:** Kentaro Matsuzaki, Naotoshi Sugimoto, Rafiad Islam, Md Emon Hossain, Eri Sumiyoshi, Masanori Katakura, Osamu Shido

**Affiliations:** 1 Department of Environmental Physiology, Faculty of Medicine, Shimane University, 89-1 Enya-cho, Izumo 693-8501, Japan; ns@med.kanazawa-u.ac.jp (N.S.); rafiad@med.shimane-u.ac.jp (R.I.); emon@uab.edu (M.E.H.); erisumi@med.shimane-u.ac.jp (E.S.); mkatakur@josai.ac.jp (M.K.); o-shido@med.shimane-u.ac.jp (O.S.); 2 Department of Physiology, Graduate School of Medical Science, Kanazawa University, 13-1 Takara-machi, Kanazawa 920-8640, Japan; 3 Department of Biotechnology and Genetic Engineering, Mawlana Bhashani Science and Technology University, Tangail 1902, Bangladesh; 4 Department of Biochemistry and Molecular Genetics, University of Alabama at Birmingham, Birmingham, AL 35294, USA; 5 Department of Nutritional Physiology, Faculty of Pharmaceutical Sciences, Josai University, 1-1 Keyakidai, Sakado, Saitama 350-0295, Japan

**Keywords:** saliva, immunoglobulin A, polymeric Ig receptor, heat acclimation, submandibular gland

## Abstract

Salivary immunoglobulin A (IgA) plays a critical role in mucosal immunity. Chronic exposure to moderate heat induces heat acclimation, which modifies salivary functions. However, the changes in salivary IgA secretion in heat-acclimated rats are unclear. In this study, we investigated salivary IgA secretion and the expression of polymeric Ig receptor (pIgR), a key mediator of mucosal IgA secretion, in the submandibular glands (SMGs) of heat-acclimated rats. Following maintenance at an ambient temperature (T_a_) of 24 ± 0.1 °C for 10 days, male Wistar rats were subjected to T_a_ of 32 ± 0.2 °C for 5 days (HE group) for heat acclimation or maintained at T_a_ of 24 ± 0.1°C (CN group). The rats were then anesthetized, pilocarpine (0.5 mg/kg) was intraperitoneally injected, and saliva was collected. Afterward, the SMGs and plasma were sampled. The salivary IgA concentration and IgA flow rate were significantly higher in the HE group than in the CN group. Similarly, SMG pIgR expression was significantly higher in HE rats. The levels of plasma cytokines, including interleukin (IL)-5, IL-6, and interferon-γ, were significantly greater in HE rats than in CN rats. Heat acclimation may enhance oral immunity through salivary IgA secretion and pIgR upregulation in the SMGs.

## 1. Introduction

Immunoglobulin A (IgA) is a type of antibody that mainly functions in the mucosal immune system and serves as the first line of defense in protecting the oral cavity and upper respiratory tract [1,2]. Because plasma cells in salivary glands produce IgA, there is a large amount of IgA in saliva [3]. The molecular mechanism of salivary IgA secretion has been studied in detail. Briefly, monomers of IgA form dimeric IgA (dIgA) through the J chain. dIgA binds the polymeric immunoglobulin receptor (pIgR) on the basolateral surface of epithelial cells and forms the IgA-pIgR complex. The IgA-pIgR complex is transported to the lumen from the basolateral surface. When pIgR reaches the apical membrane, proteolytic cleavage occurs at the apical surface. A fragment of pIgR becomes a secretory component (SC) that binds dIgA. In this manner, secretory IgA (sIgA) combines with other SCs, and free SCs are released. As a result, sIgA binds to luminal bacteria and prevents them from accessing the epithelial surface [4,5]. Therefore, a reduction in salivary sIgA levels grants bacteria access to the epithelial surface and leads to various diseases such as upper respiratory tract infection and periodontal disease [6,7].

Most studies to date have focused on the critical role of pIgR as a key mediator of mucosal IgA secretion in humans and rodents [5]. Transcriptional regulation of pIgR is modulated by multiple factors, e.g., cytokines, hormones, and bacterial products, which activate innate immunity [4,5]. In particular, the primary regulators of pIgR expression are immune system cytokines, such as interleukin (IL)-1β, IL-4, IL-5, IL-6, interferon-γ (IFNγ), and tumor necrosis factor-α (TNFα) [8,9,10,11]. Hormones such as glucocorticoids (GCs) also regulate pIgR expression [12]. Moderate exercise increases IgA secretion and the expression level of pIgR in the submandibular glands (SMGs) [13,14]. In addition, enhanced susceptibility to *Salmonella* and *Giardia* infection and increased mortality have been reported in pIgR knockout (KO) mice [15,16].

During increases in ambient temperature (T_a_) or core body temperature (T_core_), rodents have been known to spread saliva on their skin, thereby counteracting a rise in their T_core_ as a substitute for sweat [17,18]. Conversely, numerous animals can adapt physiologically and biochemically when chronically exposed to moderate heat. This process, named heat acclimation, is known to increase endurance during acute heat stress [19,20,21,22,23]. In heat-acclimated rats, the functional and morphological changes of the SMG during thermoregulation have been studied in detail [24,25,26,27]. However, functional changes in oral immunity, namely salivary sIgA secretion and pIgR expression in the SMGs of heat-acclimated rats, are unclear. Therefore, this study investigated whether heat acclimation changes salivary IgA secretion and pIgR expression in the SMGs of rats. 

## 2. Results

### 2.1. T_core_ and Locomotor Activity

**Figure 1:**
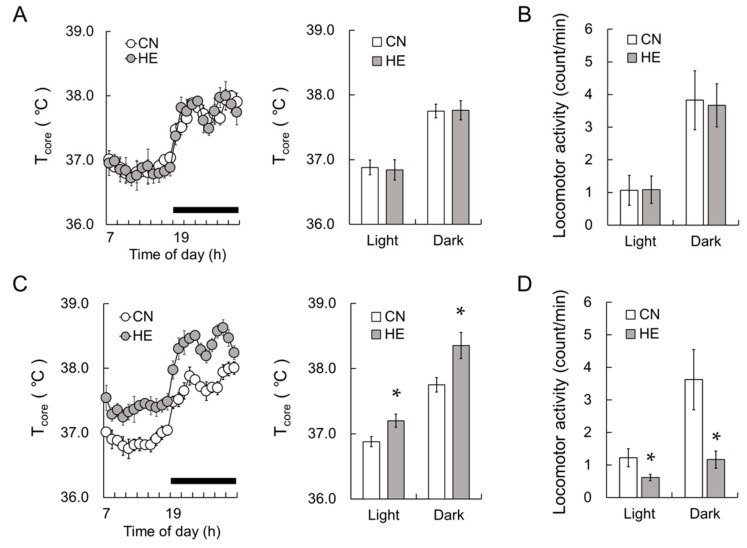
The core body temperature (T_core_) and locomotor activity of control (CN) and heat-exposed (HE) rats. (**A**) The left graph shows T_core_ of CN (open circle) and HE (gray circle) rats measured 1 day before the heat exposure period. The right graph presents mean T_core_ in the light (Light) and dark phases (Dark) in the CN (open column) and HE groups (gray column). (**B**) Locomotor activity of CN (open column) and HE rats (gray column). T_core_ and locomotor activity for (**A**) and (**B**) were measured 1 day before heat exposure. (**C**) The left graph indicates T_core_ in the CN and HE groups during heat exposure. The right graph presents mean T_core_ in the light and dark phases in the CN (open column) and HE groups (gray column) during heat exposure. Dark bars above the abscissa indicate the dark phase data. (**D**) Locomotor activity in the CN (open column) and HE groups (gray column). T_core_ and locomotor activity for (**C**) and (**D**) were measured on 2nd to 5th day of heat exposure and summarized for 24 hours. Values are presented as the mean ± SEM (*n* = 8 in each group). * *p* < 0.05, significant difference between the CN and HE groups.

Before starting heat exposure, we first observed that T_core_ did not differ between control (CN) and heat-exposed (HE) rats (Figure 1A). Mean T_core_ in the light and dark phase of CN and HE also did not differ between the groups (Figure 1A, light phase, *p* = 0.80; dark phase, *p* = 0.80). As shown in Figure 1B, locomotor activity did not differ between the groups before heat exposure in both the light and dark phases (Figure 1B, light phase, *p* = 0.80; dark phase, *p* = 0.57). Heat exposure significantly increased T_core_ (Figure 1C) in both the light (*p* < 0.05) and dark phases (*p* < 0.05). During heat exposure, T_core_ was consistently higher in the HE group than in the CN group (Supplementary Figure S1). Conversely, heat exposure decreased locomotor activity (Figure 1D) in both the light (*p* < 0.05) and dark phases (*p* < 0.05). 

### 2.2. Body, SMG, and Adrenal Gland (AG) Weight

After the heat exposure period, body, SMG, and AG weight were measured in the CN and HE groups as summarized in Table 1. The SMG and AG weights were normalized on the body weight (g) of same rats. AG weight was measured as a stress marker [28]. Heat exposure had no significant effects on their weight, although AG weight was slightly higher in the HE group than in the CN group (Table 1). 

### 2.3. Blood Cell Counts

White blood cell (WBC), red blood cell (RBC), and platelet counts (PLT), hemoglobin (HGB), hematocrit (HTC), mean corpuscular hemoglobin (MCH) levels and mean corpuscular hemoglobin concentration (MCHC) data for the two groups are summarized in Table 2. No significant differences for any of these blood components were noted between the CN and HE groups (Table 2).

### 2.4. IgA Concentration in the Saliva, SMGs and Plasma

**Figure 2:**
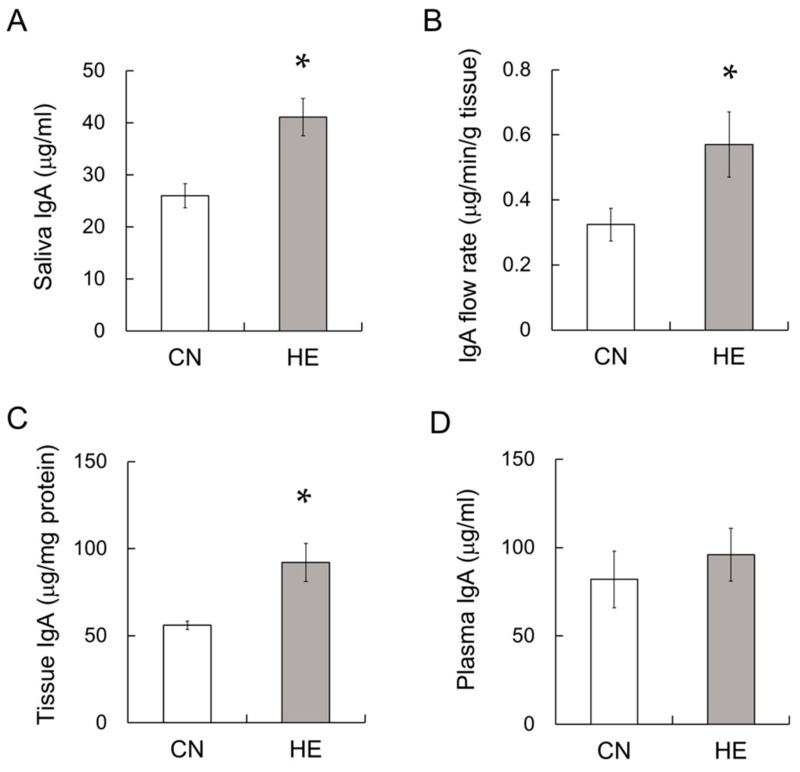
Salivary immunoglobulin A (IgA), IgA flow rate, IgA in the submandibular glands (SMGs) and plasma IgA levels in control (CN) and heat-exposed (HE) rats. (**A**) IgA concentration in saliva. (**B**) IgA flow rate. (**C**) IgA concentration of the SMGs and (**D**) plasma. Values are presented as the mean ± SEM (*n* = 8 in each group). * *p* < 0.05, significant difference between the CN and HE groups.

IgA levels in the saliva, SMG tissue, and plasma were measured via enzyme-linked immunosorbent assay (ELISA). The salivary IgA concentration was significantly different between the CN and HE groups (Figure 2A, *p* < 0.05). The IgA flow rate was significantly higher in the HE group than the CN group (Figure 2B, *p* < 0.05). The SMG IgA concentration was also significantly higher in the HE group (Figure 2C, *p* < 0.05), whereas no difference in the plasma IgA concentration was observed between the groups (Figure 2D, *p* = 0.80).

### 2.5. pIgR Expression in the SMGs

**Figure 3:**
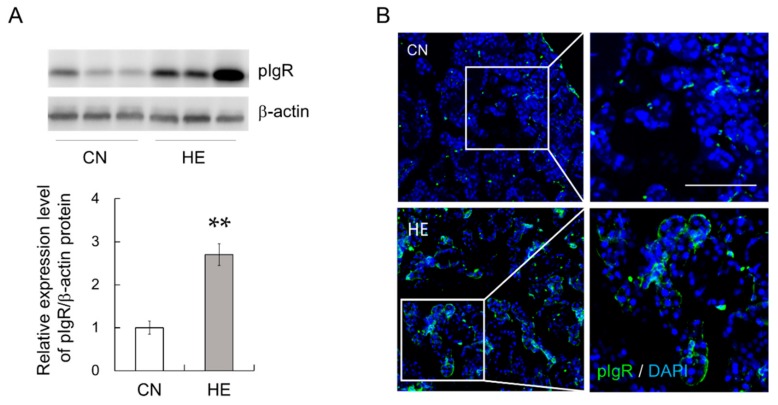
Polymeric immunoglobulin receptor (pIgR) expression in the submandibular glands (SMGs) of control (CN) and heat-exposed (HE) rats. (**A**) pIgR protein expression in the SMGs. Heat exposure increased pIgR protein expression in the SMGs. Left 3 lanes show CN rats, and right 3 lanes show HE rats. Values are presented as the mean ± SEM (*n* = 8 in each group). ** *p* < 0.01, significant difference between the CN and HE groups. (**B**) Immunohistochemical analysis of pIgR (green) in the SMGs. The nuclei were stained with 4′,6-diamidino-2-phenylindole (DAPI, blue). The right panel shows magnified views of the boxed regions from the CN and HE groups. Scale bar, 25 µm.

To test whether pIgR expression is modulated by heat exposure, we examined pIgR protein expression in the SMGs. We examined pIgR protein expression in the SMGs via Western blotting using an anti-pIgR antibody. pIgR protein expression was significantly higher in the HE group than in the CN group (Figure 3A, *p* < 0.05). Immunohistochemical analysis illustrated that pIgR was expressed in demilune structures in the salivary gland and weakly expressed in serous acinar cells, as described previously [29], and pIgR expression was markedly upregulated in the HE group compared with that in the CN group (Figure 3B).

### 2.6. Plasma Cytokine Levels

**Figure 4:**
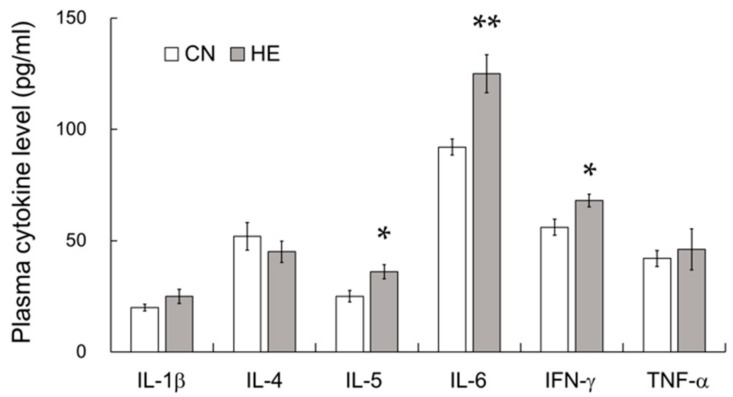
Plasma cytokine levels of control (CN) and heat-exposed (HE) rats. Plasma interleukin (IL)-1β, IL-4, IL-5, IL-6, interferon-γ (IFNγ), and tumor necrosis factor-α (TNFα) levels in the CN and HE groups. Plasma IL-5, IL-6, and IFNγ expression was significantly higher in the HE group than in the CN group. Values are presented as the mean ± SEM (*n* = 8 in each group). * *p* < 0.05, ** *p* < 0.01, significant difference between the CN and HE groups.

pIgR expression is critically regulated by cytokines, such as IL-1β, IL-4, IL-5, IL-6, IFNγ, and TNFα [8,9,10,11]. To elucidate the mechanism regulating pIgR upregulation in the SMGs, plasma cytokine levels including IL-1β, IL-4, IL-5, IL-6, IFNγ, and TNFα were measured in both groups. Plasma IL-5, IL-6, and IFNγ levels were significantly higher in the HE group than in the CN group, whereas IL-1β, IL-4, TNFα levels were not significantly changed by heat exposure (Figure 4). We could not detect any cytokines in the saliva of both CN and HE rats.

### 2.7. Plasma GC and GC Receptor (GR) Levels in the SMGs

**Figure 5:**
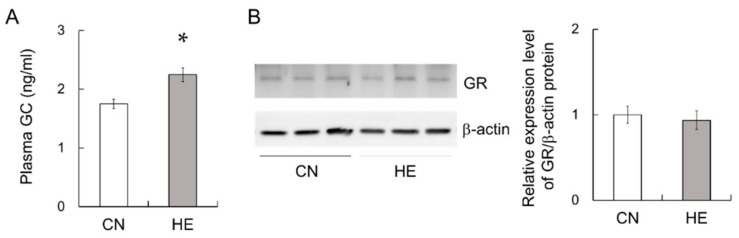
Plasma glucocorticoid (GC) level and glucocorticoid receptor (GR) expression in the submandibular glands (SMGs) of control (CN) and heat-exposed (HE) rats. (**A**) Plasma GC level in the CN and HE groups. Heat exposure significantly increased the plasma GC level in the rats. (**B**) GR protein expression in the SMGs. Heat exposure did not change GR protein expression level in the SMGs. Left 3 lanes show CN rats, and right 3 lanes show HE rats. *p < 0.05, significant difference between the CN and HE groups. Values are presented as the mean ± SEM (*n* = 8 in each group).

It has been reported that the 5′-flanking region of the *pigr* gene contains a response element to GCs [12]. This class of hormones, which includes corticosterone, is known to increase the expression of pIgR [4]. Thus, plasma GC levels were measured in CN and HE rats using ELISA. The plasma GC level was significantly higher in the HE group than in the CN group (Figure 5A), whereas GR protein level in the SMGs was not changed by heat exposure (Figure 5B). 

### 2.8. Syndecan-1 (SDC-1) Expression in the SMGs

**Figure 6:**
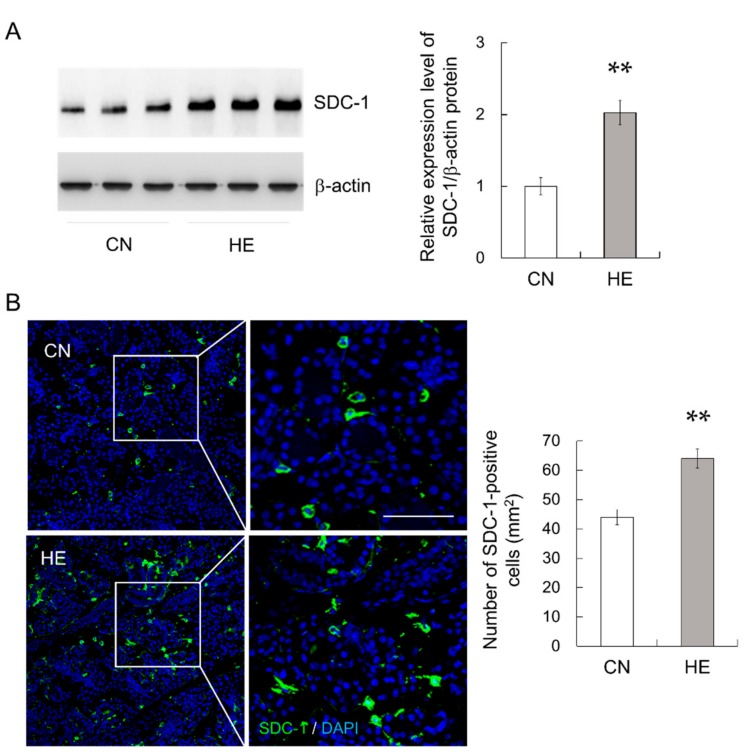
Syndecan-1 (SDC-1) expression in the submandibular glands (SMGs) of control (CN) and heat-exposed (HE) rats. (**A**) SDC-1 protein expression in the SMGs. Heat exposure increased SDC-1 protein expression in the SMGs. Left 3 lanes show CN rats and right 3 lanes show HE rats. (**B**) Immunohistochemical analysis of SDC-1 (green) in the SMGs. The nuclei were stained with 4′,6-diamidino-2-phenylindole (DAPI, blue). The right panel shows magnified views of the boxed regions from the CN and HE groups. Right graph shows the density of SDC-1-immunopositive cells in the SMG sections. Values are presented as the mean ± SEM (*n* = 8 in each group). ** *p* < 0.01, significant difference between CN and HE groups. Scale bar, 25 μm.

SDC-1, also known as CD138, is a useful marker for plasma cells because it is expressed in the late stages of B-cell differentiation with progression toward plasma cells [30]. Because plasma cells produce IgA in the salivary glands [3], we analyzed SDC-1 expression in the SMGs. SDC-1 protein expression in the SMGs was significantly higher in the HE group than in the CN group (Figure 6A, *p* < 0.01). The immunohistochemical analysis detected SDC-1 in plasma cells and a few epithelial cells exhibited cytoplasmic staining (Figure 6B). The number of SDC-1-positive cells was counted in randomly chosen high-power fields in each tissue and expressed as the number of cells per mm^2^. The number of SDC-1-positive cells in the SMGs was significantly higher in the HE group than in the CN group (Figure 6B, *p* < 0.01). In addition, many SDC-1-immunopositive cells were co-labeled with anti-IgA antibody (Supplementary Figure S2).

### 2.9. Time Course Analysis of the Effect of Heat Exposure on IgA Secretion

**Figure 7:**
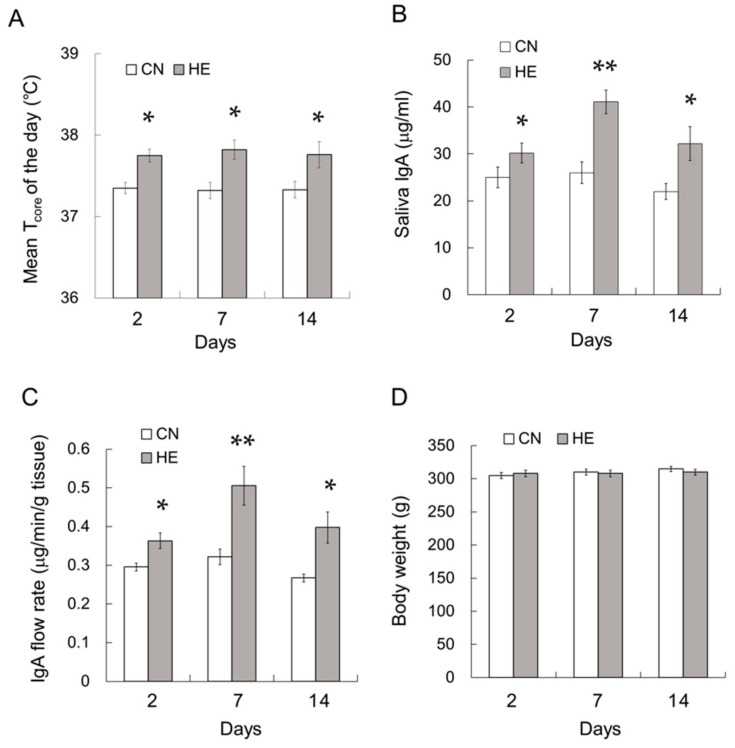
Time course analysis of the mean core body temperature (T_core_), saliva IgA concentration, IgA flow rate, and pIgR expression in the submandibular glands (SMGs) of control (CN) and heat-exposed (HE) rats. (**A**) Mean T_core_ of CN and HE rats during the heat exposure period. T_core_ was measured at 2^nd^, 7^th^ and 14^th^ day of heat exposure period. (**B**) Saliva IgA concentration. (**C**) Saliva IgA flow rate. The mean T_core_, IgA concentration and IgA flow rate were increased during the first 2 days of heat exposure and appeared to persist for at least 14 days. (**D**) Body weight of CN and HE rats. Values are presented as the mean ± SEM (*n* = 4 in each group). * *p* < 0.05, ** *p* < 0.01, significant difference between the CN and HE groups.

In a different series of the experiments, we additionally investigated that time-lapse of IgA secretion following 2, 7 and 14 days heat exposure. Before heat exposure, there were no significant differences in mean T_core_ between the groups. During the 2, 7 and 14 days heat exposure period, T_core_ in HE2, HE7, and HE14 rats were constantly higher than those in CN2, CN7, and CN14 rats, respectively (Figure 7A, *p* < 0.05). The salivary IgA concentration (Figure 7B, *p* < 0.05) and IgA flow rate (Figure 7C, *p* < 0.05) were increased during the first 2 days of the heat exposure period and remained elevated for at least 14 days. Body weight of HE14 rats was slightly lower than that of CN14, albeit without significance (Figure 7D, *p* = 0.232).

## 3. Discussion

In this study, we found that the salivary IgA concentration and IgA flow rate were significantly increased in heat-acclimated rats. The expression of pIgR, a transcytosis regulator for IgA, was also markedly enhanced in the SMGs of HE rats. Salivary IgA secretion increased in the first 2 days of the heat exposure period and remained elevated for at least 14 days. These results suggest that salivary IgA secretion may be promoted by pIgR upregulation in the SMGs of heat-acclimated rats. It is well known that the transcription of pIgR is critically regulated by cytokines [8,9,10,11]. We therefore investigated whether heat acclimation changes plasma cytokine levels in rats. IL-5, IL-6, and IFNγ levels were significantly increased in the plasma of heat-acclimated rats compared with those in CN rats, whereas plasma IL-1β, IL-4 and TNFα expression was unchanged. Changes in cytokine levels in HE rats may be involved in the changes of pIgR expression in the SMGs and IgA secretion. Given that heat stimulation induces IL-6 upregulation in the muscle [31,32], the source of plasma cytokine upregulation in HE rats may at least partly muscle-derived. It may be important to examine cytokine expression of the SMG, muscle and whole-body organs in heat-acclimated rats. We also found that the expression of SDC-1 was upregulated in the SMGs of HE rats. In addition, many SDC-1–immunopositive cells were co-labeled with IgA antibody. Because plasma cells in the salivary glands produce IgA [3], the upregulation of SDC-1 in HE rats may induce the increase of IgA production and secretion. Although the detailed mechanism of SDC-1 upregulation in the SMG is unknown, elevated cytokine may be involved in the promotion of B-cell differentiation to plasma cells. For example, IL-6 is well known to promote B-cell differentiation into plasma cells [33]. Further investigation of the effects of heat stimulation on B-cell differentiation into plasma cells is required. 

Meanwhile, continuous exposure to moderate heat (32 °C) could represent a mild stress in rats. Stress exposure activates a variety of physiological coping systems including the hypothalamic–pituitary–adrenal (HPA) axis [28]. Adrenal GCs released during stress, such as corticosterone, induce HPA axis activation and exert profound effects on immune functioning [28]. Stress responses typically promote IgA secretion, among other short-term body defense systems [34,35]. Investigations into immune changes in response to stress commonly employ sIgA as a marker of immune activation [36,37]. In this study, the plasma GC level was significantly higher in the HE group than in the CN group, whereas GR protein expression was not modulated. The AG wet weight was slightly higher in the HE group, albeit without significance. Because GC is involved in the regulation of pIgR transcription [12], the promotion of IgA secretion and pIgR expression in heat-acclimated rats might depend on both increased cytokine expression and GC production. It may be necessary to verify IgA secretion following moderate heat exposure in adrenalectomized rats. However, severe stress typically leads to deleterious health consequences, including decreases in sIgA levels [38]. It has also been reported that exposure to intense heat (40 °C) reduces intestinal IgA secretion and induces mucosal immune dysfunction in rats [39], suggesting that the promotion or suppression of IgA secretion depends on the level of heat stress. Changes in the ratio of salivary IgA secretion under different T_a_ conditions should be studied in the future.

Salivary IgA secretion is rhythmically controlled by sympathetic nerve activation by the suprachiasmatic nucleus, which functions as the main oscillator of circadian rhythms [40,41]. The circadian rhythms of pIgR expression and IgA secretion in mice peaked during the light period, and the circadian control of salivary IgA secretion vanished in *clock* KO mice [41]. In the present study, the secreted saliva of both CN and HE rats was consistently collected in the light phase. In addition, heat exposure did not affect the daily T_core_ variation (rhythm) of rats. However, it may be required to explore whether heat exposure affects clock gene expression of the SMGs in a future.

In the oral cavity, heat shock protein 70 (HSP70) has a role of mucosal defense including entrapping, agglutinating, and opsonizing bacteria and inhibiting pathogenic adhesion to the mucosal surface [42,43,44]. Salivary HSP70 binds both gram-positive (*Streptococcus mutans* and *Streptococcus mitis*) and gram-negative (*Escherichia coli*) bacteria [42,43,45]. It was also reported that HSP70 in saliva is largely produced by the SMGs, mucosal cells, and periodontal tissues [42,44]. In salivary glands and periodontal tissues, epithelial cells and myoepithelial cells express HSP70, whereas HSP70 is not expressed in acinic cells [44,46]. We have previously reported that HSP70 protein in the SMGs is significantly upregulated in heat-acclimated rats [27]. We therefore investigated whether heat acclimation modifies the expression level of HSP70 in saliva. Our preliminary survey revealed that salivary HSP70 expression was significantly elevated in heat-acclimated rats (Supplementary Figure S3). Heat exposure may strengthen oral immunity by both increasing IgA secretion and enhancing HSP70 secretion in the saliva. Further investigations may be required to clarify the exact source of salivary HSP70 in heat-acclimated rats and whether elevated HSP70 expression in saliva and SMGs in heat-acclimated animals contributes to preventing oral infections.

## 4. Materials and Methods

### 4.1. Ethics Statement

All animal experiments in this study were performed in accordance with the Guidelines for Animal Experimentation of the Shimane University Faculty of Medicine in compliance with the Guidelines for Animal Experimentation of the Japanese Association for Laboratory Animal Science. The protocol for this study was approved by the Committee on the Ethics of Animal Experiments of Shimane University (Approval number: IZ30-56).

### 4.2. Experimental Schedule

Sixteen male 10-week-old Wistar rats (Japan SLC Inc., Hamamatsu, Japan) were maintained for 7 days at T_a_ of 24.0 ± 0.1 °C and relative humidity of 45% ± 5% under a 12-h:12-h light–dark cycle (light phase, 07:00–19:00) with food and water provided ad libitum. After 7 days post-arrival of the animals at the facility, rats were anesthetized using a combination anesthetic containing 0.15 mg/kg medetomidine (Kyoritsu Seiyaku, Tokyo, Japan), 2.0 mg/kg midazolam (Astellas Pharma, Tokyo, Japan), and 2.5 mg/kg butorphanol (Meiji Seika Pharma, Tokyo, Japan). Then, a TA10TA-F40 temperature transmitter (Data Sciences International, St Paul, MN, USA) was implanted in each rat’s intraperitoneal cavity. Rats were allowed to recover from surgery for 14 days prior to data collection. After the recovery period, rats in the heat acclimation group (HE, *n* = 8) were subjected to a constant T_a_ of 32.0 ± 0.2 °C for 5 days, whereas control rats (CN, *n* = 8) were continuously maintained at 24.0 ± 0.1 °C, as described previously [47,48,49]. One day before, and during 2^nd^ to 5^th^ days of heat exposure period, T_core_ and locomotor activity were measured using a telemetry system [50,51]. 

### 4.3. Saliva Collection

After the heat exposure period, saliva secretion induced by pilocarpine (Fujifilm Wako Pure Chemical, Tokyo, Japan) was collected as described previously [52]. Briefly, rats were weighed and anesthetized at approximately 10:00 h (light phase), a cotton ball was placed in their mouths sublingually, and pilocarpine (0.5 mg/kg) was intraperitoneally injected to induce saliva secretion. Pilocarpine, an M3 muscarinic receptor agonist, has been proven to be useful for inducing and assessing saliva secretion [52,53]. The cotton ball was then changed every 10 min for 1 h. The 6 cotton balls collected from each rat were centrifuged, and saliva was collected. 

### 4.4. Blood and Tissue Collection

After saliva collection, blood (approximately 4 mL) was collected from the right ventricle of rats while anesthetized and placed into a sterile tube containing heparin sodium (Mochida Pharmaceutical Co., Ltd, Tokyo, Japan). Then, saline was transcardially perfused, and the SMGs and AGs were sampled and weighed. The right SMG was divided into two pieces and the entire half was used for immunohistochemistry and the rest for Western blotting and ELISA. The SMG sample for Western blotting and ELISA was flash frozen in liquid nitrogen and stored at −80 °C until use. The SMG for immunohistochemistry was fixed overnight in Mildform 10N (Fujifilm Wako Pure Chemical) at 4 °C and immersed overnight in a 20% (*w*/*v*) sucrose solution. Then, the SMGs were fixed in OCT compound (Sakura Finetek Japan Co., Ltd., Tokyo, Japan) and stored at −30 °C until use.

### 4.5. Blood Cell Counts

After blood collection, 0.5 mL of blood sample was immediately used for blood cell counts. The following variables were measured in the CN and HE groups using a KX-21NV automatic hemocytometer (Sysmex, Kobe, Japan) as described previously [52,54]: WBC, RBC, PLT, HGB, HTC, MCH, MCHC and MCV. After blood cell counts, approximately 3.5 ml of blood was centrifuged (1500× *g*) for 20 min at 4 °C. Plasma was collected, flash frozen in liquid nitrogen and stored at −80 °C until use.

### 4.6. Western Blot Analysis

The SMGs were homogenized using a glass homogenizer in lysis buffer containing 150 mM sodium chloride, 1% Triton X-100, 0.1% sodium dodecyl sulfate (SDS), 1× protease inhibitor cocktail (Fujifilm Wako Pure Chemical), and 10 mM Tris-HCl (pH 7.6). After sonication and removal of the tissue debris via centrifugation at 10,000× *g* for 15 min at 4°C, the supernatants were analyzed via Western blotting as described previously [52]. Briefly, the concentrations of proteins extracted from the SMGs were determined using a Pierce BCA protein assay kit (Thermo Fisher Scientific, Waltham, MA, USA). Equal amounts of protein extracts were boiled in 6 × SDS sample buffer (Nacalai Tesque, Kyoto, Japan). Samples were separated via 12.5% SDS–polyacrylamide gel electrophoresis. The resolved proteins were transferred onto polyvinylidene fluoride (PVDF) membranes (Millipore, Billerica, MA, USA) blocked with 5% skimmed milk and then incubated with primary antibodies, namely polyclonal rabbit anti-pIgR (1:1000; GeneTex Irvine, CA, USA), polyclonal rabbit anti-syndecan-1 (1:1000; BioVision, Milpitas, CA, USA), or monoclonal mouse anti-glucocorticoid receptor (1:1000; GeneTex), at 4 °C for 12 h. After washing, the PVDF membranes were incubated with horseradish peroxidase-linked anti-mouse, anti-rabbit, or anti-goat secondary antibodies (1:2000; Cell Signaling, Danvers, MA, USA) at room temperature for 2 h. The blots were developed using SuperSignal™ West Pico PLUS Chemiluminescent Substrate (Thermo Fisher Scientific) and visualized using a LAS 4000 visualizer imaging system (Fujifilm, Tokyo, Japan). The membranes were then stripped and reprobed with monoclonal rabbit anti-β-actin antibody (1:5000; Cell Signaling, Danvers, MA, USA) to ensure that equal amounts of protein were loaded. Each protein band was quantitated by imaging software Multi gauge Version 3.0 (Fujifilm), and the target protein was normalized on the β-actin expression of the same sample.

### 4.7. ELISA

The IgA concentration in saliva and SMG samples was measured using an IgA ELISA Kit (Abnova, Taipei, Taiwan) according to the manufacturer’s protocol. The plasma GC concentration was measured using a General Glucocorticoid ELISA Kit (MyBioSource, San Diego CA, USA). Saliva HSP70 concentration was measured using a HSP70 ELISA kit (StressMarq Biosciences Inc. Victoria, British Columbia, Canada). Absorbance was measured using a DTX880 multi-mode microplate reader (Beckman Coulter, Pasadena, CA, USA). IgA, GC and HSP70 concentrations were calculated using SoftMax pro software (Molecular Devices, LLC, San Jose, CA, USA) as described previously [54,55]. The IgA flow rate in saliva (μg/min/g SMG tissue) was calculated by multiplying the absolute concentration of IgA (μg/mL) by the saliva flow rate (mL/min) as per g of SMG tissue.

### 4.8. Immunohistochemistry 

A CM1520 cryostat (Leica, Wetzlar, Germany) was used to prepare 15-µm-thick SMG sections, which were then incubated in 10 mM sodium citrate buffer (pH 6.0) and blocked with 3% normal goat serum (Agilent, Santa Clara, CA, USA). For multiplex immunoassaying, the SMG sections were incubated with primary antibodies at 4 °C for 12 h. The primary antibodies used in this study were polyclonal rabbit anti-pIgR (1:500), polyclonal goat anti-IgA (1:500, Novus Biologicals, LLC, Centennial, CO, USA), and monoclonal mouse anti-SDC-1 (1:500). Alexa Fluor 488-conjugated anti-mouse IgG (1:500; Molecular Probes, Waltham, MA, USA), Alexa Fluor 488-conjugated anti-rabbit IgG and Alexa Fluor 633-conjugated anti-goat IgG (1:500; Molecular Probes) were used as the secondary antibodies. To detect cell nuclei, sections were counterstained with 4′,6-diamidino-2-phenylindole solution (DAPI, 1:2000, Dojindo, Kumamoto, Japan). After staining, the sections were washed and covered with 80% glycerol. An FV-1000D confocal microscope (Olympus, Tokyo, Japan) and Fluoview imaging software (Olympus) were used to visualize all sections under ×20 or ×40 magnification, as described previously [52]. 

### 4.9. Cytokine Measurements 

Plasma and saliva samples were assayed for cytokine concentrations using multiplexed (IL-1β, IL-4, IL-5, IL-6, IFNγ, and TNFα) bead-based immunoassay kits combined with a Cytokine Reagent Kit (Bio-Rad Laboratories, Hercules, CA, USA) and a Bio-Plex™ Diluent Kit (Bio-Rad Laboratories) in the Bio-Plex™ MAGPIX System (Bio-Rad Laboratories) according to the manufacturer’s protocol. Concentrations of plasma cytokines were calculated using Bio-Plex™ Manager MP Software (Bio-Rad Laboratories).

### 4.10. Time Course Effects of Heat Exposure on IgA Secretion

Additionally, 24 male Wistar rats (10 weeks old) were used to perform time-lapse analysis of IgA secretion following heat exposure. Rats were maintained for 7 days at T_a_ of 24.0 ± 0.1 °C and relative humidity of 45% ± 5% under a 12-h:12-h light–dark cycle with food and water provided ad libitum. Then, all rats were anesthetized, and a telemetry transmitter was implanted into their abdominal cavities. After a 14-day recovery period, rats in the HE group were subjected to a constant T_a_ of 32.0 ± 0.2 °C and relative humidity of 45% ± 5%, whereas CN rats were continuously kept at 24.0 ± 0.1 °C. On the 2nd (HE2), 7th (HE7), and 14th (HE14) days of the heat exposure period (*n* = 4 in each group), the rats were weighed and anesthetized, pilocarpine (0.5 mg/kg) was injected, and saliva was sampled as described previously. The same procedure was applied to control rats without heat exposure, i.e., the saliva was collected on the 2nd (CN2), 7th (CN7), and 14th (CN14) days (*n* = 4 in each group). Salivary IgA levels and the IgA flow rate were measured as described previously.

### 4.11. Data Quantification and Statistical Analysis

The results are presented as the mean ± SEM. Statistical analyses were performed using SPSS software version 22.0 (IBM Corp., Armonk, NY, USA). The Mann–Whitney *U* test was used for comparisons between 2 groups. Analysis of variance followed by Bonferroni’s post hoc test was used to examine any significant group differences in time-lapse analysis of the effect of heat exposure on mean daily T_core_, salivary IgA concentrations, IgA flow rates, and body weights. *p* < 0.05 denoted statistical significance.

## 5. Conclusions

The results of this study demonstrated that constant exposure to moderate heat facilitated salivary IgA secretion and upregulated pIgR expression in the SMGs of rats. Although further research is required to elucidate the mechanism, heat acclimation enhances oral immune functions, and it may be beneficial for preventing upper respiratory tract infection and periodontal disease. Because body temperature and immune function are closely involved [56], it may be important to examine the immune function of both the salivary gland and whole-body organs in heat-acclimated animals.

## Figures and Tables

**Table 1:** Body weight (BW), submandibular gland (SMG) and adrenal gland (AG) weight of the control (CN) and heat-exposed (HE) rats.

	CN	HE	*P* Value
BW (g)	310.7 ± 3.2	305.5 ± 3.9	0.083
SMG/BW (mg/g)	0.98 ± 0.01	1.00 ± 0.02	0.505
AG/BW (mg/g)	0.15 ± 0.01	0.16 ± 0.01	0.442

There was no significant difference in BW, SMG/BW and AG/BW between the CN and HE groups. The SMG and AG weights were normalized on the BW of same rats. Values are presented as the mean ± SEM (*n* = 8 in each group).

**Table 2:** Blood cell components for the control (CN) and heat-exposed (HE) rats.

	CN	HE	*P* Value
WBC (× 10^2^/μL)	66.7 ± 3.2	68.5 ± 3.9	0.130
RBC (× 10^5^/μL)	86.0 ± 3.6	87.5 ± 4.6	0.579
PLT (× 10^4^/μL)	68.4 ± 2.4	65.7 ± 4.8	0.234
HGB (g/dL)	15.1 ± 1.1	15.5 ± 0.8	0.505
HTC (%)	48.0 ± 2.8	50.2 ± 3.3	0.161
MVC (fl)	54.9 ± 1.2	55.4 ± 1.5	0.195
MCH (pg)	17.5 ± 1.0	18.0 ± 1.2	0.234
MCHC (g/dL)	30.2 ± 2.0	31.8 ± 2.1	0.195

WBC, white blood cell; RBC, red blood cell; PLT, platelets; HGB, hemoglobin; HTC, hematocrit; MCV, mean corpuscular volume; MCH, mean corpuscular hemoglobin; MCHC, mean corpuscular hemoglobin concentration. There was no significant difference in any blood cell component between the CN and HE groups. Values are presented as the mean ± SEM (*n* = 8 in each group).

## Supplementary Materials

The following are available online at https://www.mdpi.com/1422-0067/21/3/815/s1, Figure S1: The T_core_ of CN and HE during heat exposure period, Figure S2: Double-staining of IgA and SDC-1 in the SMG of HE rat, Figure S3: Salivary HSP70 expression of CN and HE groups.

## Abbreviations

AG	Adrenal gland
CN	Control
ELISA	Enzyme-linked immunosorbent assay
GC	Glucocorticoid
GR	Glucocorticoid receptor
HE	Heat-exposed
HGB	Hemoglobin
HTC	Hematocrit
IFNγ	Interferon-γ
IgA	Immunoglobulin A
IL	Interleukin
MCH	Mean corpuscular hemoglobin
MCHC	Mean corpuscular hemoglobin concentration
MCV	Mean corpuscular volume
pIgR	Polymeric immunoglobulin receptor
PLT	Platelet
RBC	Red blood cell
SC	Secretory component
SMG	Submandibular gland
TNFα	Tumor necrosis factor-α
WBC	White blood cell
