# Supplementary material for: Salivary Immunoglobulin A Secretion and Polymeric Ig Receptor Expression in the Submandibular Glands Are Enhanced in Heat-Acclimated Rats
Source: Int J Mol Sci. 2020 Jan 27;21(3):815. doi: 10.3390/ijms21030815 (PMC7037029; doi:10.3390/ijms21030815)
Supplement: Supplementary file 1 [file ijms-21-00815-s001.pdf]

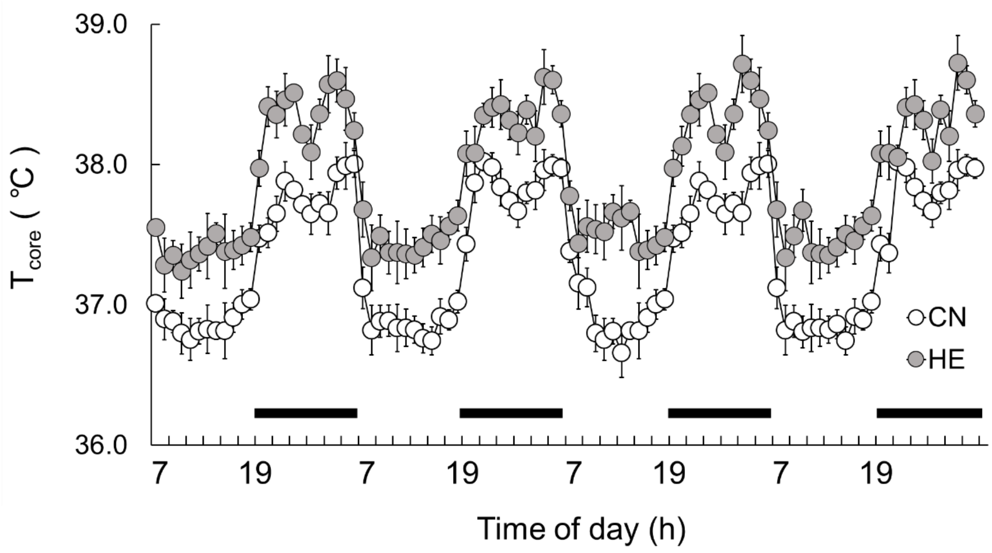

Figure S1. The core body temperature (T_{core}) of control (CN, open circle) and heat-exposed rats (HE, grey circle) during heat exposure period. Values are the means ± SEMs. Dark bars above abscissa indicate the dark phase of the day.

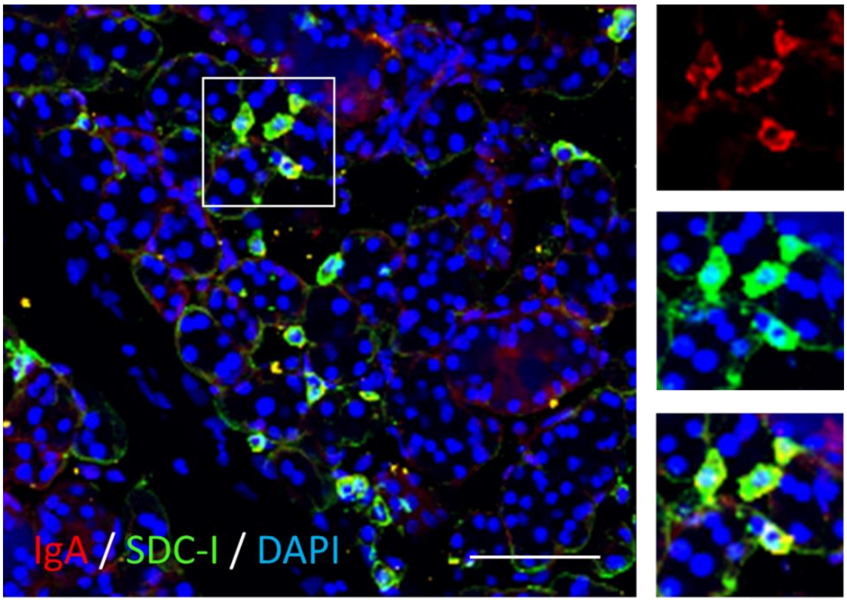

Figure S2. Double-staining of Immunoglobulin A (IgA) and Syndecan-I (SDC-1) in the submandibular gland (SMG) of heat-exposed rat. SDC-1–immunopositive cells (green) were frequently co-stained with anti-IgA antibody (red) in the SMGs. The nuclei were counter-stained with 4′,6-diamidino-2-phenylindole (DAPI, blue). The right panel shows magnified views of the boxed regions in left. Scale bar, 25 μm.

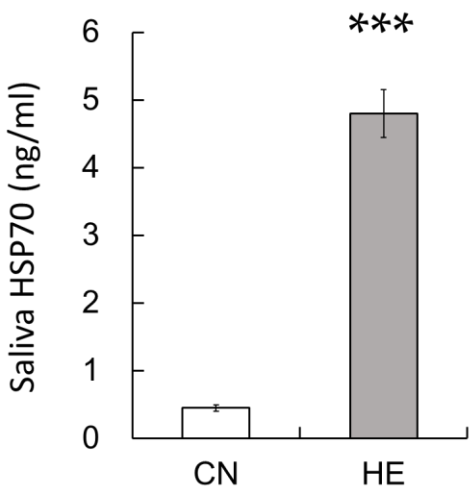

Figure S3. Salivary heat shock protein 70 (HSP70) expression of control (CN, open column) and heat-exposed rats (HE, grey column). Values are the means ± SEM. ***$p < 0.001$, significant difference between the CN and HE groups.
